# Practical Clinical and Diagnostic Pathway for the Investigation of the Infertile Couple

**DOI:** 10.3389/fendo.2020.591837

**Published:** 2021-01-19

**Authors:** Andrea Garolla, Damiano Pizzol, Andrea Roberto Carosso, Andrea Borini, Filippo Maria Ubaldi, Aldo Eugenio Calogero, Alberto Ferlin, Antonio Lanzone, Francesco Tomei, Bruno Engl, Laura Rienzi, Lucia De Santis, Giovanni Coticchio, Lee Smith, Rossella Cannarella, Attilio Anastasi, Massimo Menegazzo, Liborio Stuppia, Christian Corsini, Carlo Foresta

**Affiliations:** ^1^ Section of Andrology and Reproductive Medicine & Centre for Male Gamete Cryopreservation, Department of Medicine, University of Padova, Padova, Italy; ^2^ Italian Agency for Development Cooperation, Public Health, Jerusalem, Israel; ^3^ Department of Surgical Sciences, Gynecology and Obstetrics 1, Physiopathology of Reproduction and IVF Unit, S. Anna Hospital, University of Torino, Torino, Italy; ^4^ 9.baby, Family and Fertility Center, Tecnobios Procreazione, Bologna, Italy; ^5^ GENERA Centre for Reproductive Medicine, Clinica Valle Giulia, Rome, Italy; ^6^ Department of Clinical and Experimental Medicine, University of Catania, Catania, Italy; ^7^ Department of Clinical and Experimental Sciences, University of Brescia, Brescia, Italy; ^8^ Department of Woman’s Health Sciences of the Child and Public Health, Unit of Obstetrics Pathology, University Clinic Foundation “A Gemelli” IRCCS, Rome, Italy; ^9^ Clinic of Obstetrics and Gynecology, Catholic University Sacro Cuore, Rome, Italy; ^10^ Assisted Reproductive Unit, Santa Maria degli Angeli Hospital, Pordenone, Italy; ^11^ Donna Salus, Center for Women’s Health and Fertility, Bolzano, Italy; ^12^ IVF Unit, Gynaecological-Obstetric Department, IRCCS San Raffaele Hospital, Vita-Salute University, Milan, Italy; ^13^ Italian Society of Embryology, Reproduction and Research (SIERR), Giarre, Italy; ^14^ The Cambridge Centre for Sport & Exercise Sciences, Anglia Ruskin University, Cambridge, United Kingdom; ^15^ Center for Physiopathology of Human Reproduction, Delta Hospital, Lagosanto, Italy; ^16^ Department of Psychological, Health and Territorial Sciences, School of Medicine and Health Sciences, “G. d’Annunzio” University, Chieti, Italy; ^17^ Center for Advanced Studies and Technology (CAST), “G. d’Annunzio” University of Chieti-Pescara, Chieti, Italy

**Keywords:** infertility diagnosis, female infertility, couple infertility, male infertility, reproduction, diagnosis, management, assisted reproductive technlogy

## Abstract

**Capsule:**

This expert opinion summarizes current knowledge on risk factors for infertility and identifies a practical clinical and diagnostic approach for the male and female partners of an infertile couple aimed to improve the investigation and management of fertility problems.

**Background:**

Infertility represents an important and growing health problem affecting up to 16% of couples worldwide. In most cases, male, female, or combined factor can be identified, and different causes or risk factors have been related to this condition. However, there are no standardized guidelines on the clinical-diagnostic approach of infertile couples and the recommendations concerning infertility are sometimes lacking, incomplete, or problematic to apply.

**Objective:**

The aim of this work is to provide an appropriate clinical and diagnostic pathway for infertile couples designed by a multidisciplinary-team of experts. The rationale is based on the history and physical examination and then oriented on the basis of initial investigations. This approach could be applied in order to reduce variation in practice and to improve the investigation and management of fertility problems.

**Methods:**

Prominent Italian experts of the main specialties committed in the ART procedures, including gynecologists, andrologists, embryologists, biologists, geneticists, oncologists, and microbiologists, called “InfertilItaly group”, used available evidence to develop this expert position.

**Outcomes:**

Starting from the individuation of the principal risk factors that may influence the fertility of females and males and both genders, the work group identified most appropriate procedures using a gradual approach to both partners aimed to obtain a precise diagnosis and the most effective therapeutic option, reducing invasive and occasionally redundant procedures.

**Conclusions:**

This expert position provides current knowledge on risk factors and suggests a diagnostic workflow of infertile couples. By using this step-by-step approach, health care workers involved in ART, may individuate a practical clinical management of infertile couples shared by experts.

## Introduction

WHO sets the clinical definition for infertility as a disease of the reproductive system defined by the failure to achieve a clinical pregnancy after 12 months or more of regular unprotected sexual intercourse (1). Although it is difficult to estimate the accurate global burden of infertility and sub fertility, they affect a significant proportion of humanity with an estimated prevalence of 72.4 million infertile people and of these 40.5 million seeking infertility medical care (2). Infertility not only is related to the reproductive health but has psychological, economic, and medical implications resulting in trauma, stress, especially in societies and cultures addressing strong emphasis on child-bearing (1). As a logical result, and attempting to find a solution, assisted reproductive technologies (ARTs) spread globally, constituting a treatment of choice for complicated fertility disorders (3). The term ART denominates all interventions, including the *in vitro* handling of human oocytes, spermatozoa and embryos, for the purpose of reproduction (3). Owing to the increasing complexity of treatment options, ART procedures are delivered *via* highly specialized health care workers including: gynecologists, andrologists, embryologists, geneticists, oncologists, urologists, psychologists, obstetricians, and others (4). Nevertheless, there are no international standardized rules and laws regulating these procedures, resulting in the worldwide spread of ART clinics having variable quality standards (4). It is estimated that more than five million infants have been born from ART and, interestingly, Europe has been the most active continent (5).

In order to reduce variation in practice and improve the way fertility problems are investigated and managed, different guidelines have been developed both at national and international levels.

The National Institute of Health and Care Excellence (NICE) guidelines make evidence-based recommendations on a wide range of topics including fertility impairment. They are probably one of the most important international clinical guidelines concerning infertility assessment and treatment. Their development follows rigorous methods and criteria to ensure reliable recommendations for good clinical practice.

The NICE guidelines on fertility examine all aspects related to fertility, from principles of care and initial advice for people concerned about delays in conception, to investigation and management of fertility problems, proper diagnosis work-up and appropriate treatment approach (https://www.nice.org.uk/guidance/cg156). However, some conditions related to age of patients, general health, risk factors, economic situation etc., can raise critical issues making NICE guidelines difficult to apply. One issue is linked to the advanced age. According to European statistical institutes (Eurostat), in 2017 the average age of women who gave birth to their first child is 31.1 years, the highest in Europe where the average is 29.1 years. This determines the need to speed up the diagnostic process of infertile women, who often come to the observation of reproductive medicine specialists at an advanced maternal age and with a compromised ovarian reserve. In addition, some countries do not comply with the NICE guidelines because they have not the possibility to perform invasive procedures within their own center or within reasonable time. Most centers, especially private ones, do not have surgery rooms capable of performing specific techniques, such as laparoscopy. Surgical procedures are often performed by surgeons who are not involved in reproductive medicine, and who therefore are not familiar with techniques such as ovarian drilling or conservative tubal surgery, which should be entrusted to teams with sufficient experience in these procedures. The aforementioned reasons limit access to the invasive diagnostic and therapeutic procedures suggested by the NICE guidelines, which should be reserved for a strictly selected group of patients in a such different context.

Another issue that limits the use of the NICE guidelines is linked to the different legislation in force, compared to most European countries. In Italy, for example, drugs such as metformin for polycystic ovary syndrome (PCOS) are still off-label, the cryopreservation of embryos is theoretically prohibited (although made possible by way of derogation from the law and with well-defined limits) and the donation of gametes is still severely limited as donors are not allowed to receive an economic fee.

The NICE guidelines also deal with male factor infertility and its medical and surgical management. On this topic, there is a great difference with the detailed analysis of female factor infertility. For instance, some important risk factors for male infertility are not mentioned (e.g. cryptorchidism, orchitis and epididymitis, testicular hypotrophy, seminal tract infections, testicular trauma or torsion, testicular neoplasia, systemic diseases, hormonal derangement due to anabolic steroid use, etc.). Moreover, minimal diagnostic work-up is not defined, other than seminal analysis. Recommendations about physical examination, hormonal tests, microbiological analysis, and imaging diagnostics are lacking.

The present expert opinion, describes a practical clinical and diagnostic pathway for infertile couples designed by a multidisciplinary team of experts in the ART procedures, named “InfertilItaly group”, including gynecologists, andrologists, embryologists, biologists, geneticists oncologists, and microbiologists. The objective of this work is to provide clear indications for the management of the infertile couple, completing the diagnostic process as accurately and quickly as possible, taking into account the Italian health, legislative, and social context.

## Methods

At the beginning of 2020, a final meeting of Italian experts in couple infertility was held in Rome to discuss risk factors and diagnostic workflow of infertile couples. This document is the result of several meetings, drafts, data discussions, and covered guidance for the care of infertile couples. The proceedings journal is published in Italian (6) and it is downloadable at https://www.ccgm.it website. The final meeting in February 2020 was organized to provide a forum for interdisciplinary discussion involving as many interested parties as possible. More than 100 participants from all Italian Regions took part in the meeting. They included all the professionals involved in the management of the infertile couple. The objectives of the meeting were a) to review literature about causes and risk factors of infertility, b) to identify unresolved issues in the field, and c) to provide standard recommendations tailored for couples seeking fertility in the Italian context.

To permit in-depth analysis and discussion, the meeting did not cover the whole field of ART. Instead, it focused on a selected number of topics including infertility risk factors, national and international surveillance of ART, diagnostic tools and procedures, psychosocial issues, ethical and law aspects in relation to the individual, to the couple and to the offspring, as well as issues of equitable access, the role of consumers.

Background papers were commissioned from invited speakers and peer-reviewed prior to the meeting. The papers were briefly presented with emphasis being given to discussion and the delineation of recommendations for practice and future research. All of the meeting sessions were plenary to allow full interaction among the different disciplines. A wide variety of views and approaches was presented in an intense, dynamic, and rich debate.

This document comprises five sections, consisting of the main specific areas discussed and the recommendations agreed to by the meeting participants: i) risk factors, ii) assessment of infertile couples, iii) infertile couple pathway, iv) gynecological specific flow charts, and v) andrological specific flow charts. The sections are grouped by expertise area in an order similar to that of the final meeting program but also in a way that the different views are juxtaposed or complementary. Despite the diverse opinions on ART voiced at the meeting, there were many points upon which a final consensus was reached.

### Risk Factors

Despite general perception that causes of infertility predominantly affect female, the causes of infertility are in fact equally distributed between the sexes. Indeed, according to the American Society for Reproductive Medicine, in 40% of couples with infertility, the female partner is either the single or a contributing cause of infertility, in 40% it is the male partner and in the remaining 20% there are no identifiable reasons termed unexplained infertility (7). Male factor infertility is defined as one or more abnormal parameters detected on semen analysis or the presence of inadequate sexual or ejaculatory function (8). The main fertility risk factors affecting males, females and both sexes are listed in **Table 1**.

**Table 1:** Female and male infertility risk factors.

Both genders	Female	Male
Family history of infertility	Age >35 years	Cryptorchidism
Recurrent abortions	Reduced ovarian reserve (AMH and/or AFC count)	Testicular hypotrophy
Obesity	Ovulation Disorders	Testicular cancer
Using of anabolic steroids	Infertility >3 years	Known genetic factors
Lifestyles	Menstrual disorders	Varicocele
Environmental/occupational factors	Endometriosis	Testicular trauma
Systemic and/or endocrine diseases	Family history of POF	Testicular torsion
Iatrogenic factors	PID (consequent to infectious diseases)	Puberty disorders
Infertility with previous partners		Aging
Cystic fibrosis		Testicular microlithiasis
Infection		Unhealthy diet and obesity Pollution Cigarette smoking

AFC, antral follicle count; AMH, anti-müllerian hormone; BMI, body max index; PID, pelvic inflammatory disease; POF, primary ovarian insufficiency.

#### Risk Factors for Both Sexes

Several common factors may affect fertility in both sexes with different impact (9). It is well known that fertility declines with age in males and females, but the decline is much faster in women. Female age is not a modifiable risk factor, this is linked both to the progressive depletion of the number of oocytes contained within the ovary (ovarian reserve), and to the progressive deterioration of their chromosomal and structural integrity (10). The increasingly advanced age at which women begin to desire a pregnancy has progressively increased the time window in which numerous lifestyle factors can exert their negative influence on the reproductive system, gametes, and general health. These lifestyle risk factors include: not achieving dietary recommendations and excessive physical activity participation (11–13); overweight (both BMI >25 and BMI >30 are related to a reduction in fertility) (14–17); physical, social or psychological stress (18–20); smoking tobacco (21–23); alcohol consumption; a high caffeine consumption; taking illicit drugs (24, 25); and a wrong timing of sexual intercourse at reproductive aim (26).

Growing evidence exists regarding the role of infectious agents on fertility impairment. In women, they can cause pelvic inflammatory disease and tubal obstruction (27) and in men they can lead to organ damage, create an obstruction or induce cell damage *via* mediators of inflammation or binding to spermatozoa (28). Interestingly, from 1993 WHO established the role of genital tract infections in human infertility and further studies reported that 15%–20% of infertile subjects are affected by semen infection (29).

#### Female Risk Factors

Female infertility may have different causes, like alterations of the reproductive system, congenital malformations, infections and hormonal dysfunctions. Particular attention should be paid to women with reduced ovarian reserve, linked to iatrogenic causes (for example previous cytotoxic treatments), or linked to a reduced follicular heritage from birth (POF family history) (10). In addition to a reduced ovarian reserve, several risk factors can affect the female fertility, and the main ones are summarized in **Table 1**. Alongside lifestyle risk factors, an important determinant of female infertility is linked to the reduction in the number of ovulatory and, therefore, potentially fertile cycles. PCOS is the most common endocrine disorder and is the leading cause of anovulatory infertility in women. It is found in 5%–10% of the female population of reproductive age (30). According to ESHRE guidelines, PCOS has to be investigated in cases of clinical or biochemical hyperandrogenism, ovulatory dysfunction, or polycystic ovaries (31). While ESHRE guidelines suggest that where irregular menstrual cycles and hyperandrogenism are present ultrasound is not necessary for diagnosis, in infertile couples it should be mandatory in order to exclude other possible causes of female infertility.

Together with PCOS, all causes of amenorrhea may be a risk factor for female infertility since they are associated with ovulatory disorders. The term amenorrhea defines the absence of spontaneous menstruation for a period of at least 90 days or, according to some classifications, at least 180 days. It is necessary to differentiate physiological amenorrhea (present in some phases of a woman’s life, such as pre-pubertal age, pregnancy, and post-menopause) from pathological amenorrhea. The prevalence of the latter is around 3%–4%. Among the various classifications, the distinction between primary amenorrhea (absence of menarche from the age of 15 in the presence of secondary sexual characteristics or, in the absence of the latter, the absence of menarche from the age of 13) and secondary (absence of menstrual cycles for a period of at least 6 months in a woman who has had previous regular menstrual cycles or at least 12 months in a woman with oligomenorrheic cycles) is considered particularly useful. Indeed, in the presence of secondary amenorrhea, there is a previous evidence of anatomical activity functional of the reproductive axis and of the internal and external genital apparatus, which on the contrary is to be proved in the case of primary amenorrhea. For a list of possible causes of amenorrhea, see the position statement of the American Society for Reproductive Medicine (32).

Endometriosis is another known factor of female infertility. Epidemiological data available in the literature, estimate that 6%–10% of the general female population suffers from endometriosis. In infertile women, the prevalence is estimated to be between 25% and 30% while 30%–50% of patients with endometriosis, independently from its stage, are infertile. Hughes et al. reported a monthly fertility rate for patients with endometriosis between 2% and 10%, considerably lower than that of the general population estimated to be between 15% and 20% (33).

Together with the above, other risk factors have been associated with female infertility, although their prevalence is lower than the previous ones.

#### Male Risk Factors

Male infertility is usually related to a reduced number of spermatozoa or to abnormalities in their quality. Two main conditions of male infertility are recognized: primary infertility, defined when the man has never induced a pregnancy and secondary infertility, when the man has previously fathered. An important risk factor is cryptorchidism. In fact, in congenital cryptorchid testes germ cell loss and the deterioration of the testicular structure can be observed. Importantly, these detrimental changes are observed to a higher level when testes remain undescended (34). Although orchiopexy corrects the inappropriate temperature exposure of the testis, surgery may not reverse the dysgenetic damage that underlies cryptorchidism in the first place (34). Another debated but recognized risk factor is varicocele that, on one side is associated with negative effects on semen quality, sperm function, testicular histology, and reproductive hormones and, on the other side it is present in men able to father children (35). Besides, patients with testicular cancer may have infertility both before cancer treatments due to systemic effects, endocrine changes, possible autoimmune effects, intrinsic testicular damage, possible congenital abnormalities in testicular maturation and after gonadotoxic effects related to orchiectomy, radiation, and chemotherapy (36). Growing evidence is also focusing on genetic factors and it is indeed well established that genetic causes account for 10%–15% of infertility cases, including chromosomal abnormalities and single-gene mutations (37). In addition to these well-known factors, growing evidence suggests that obesity, unhealthy diet, cigarette smoking and pollution may seriously affect sperm parameters and reduce male fertility (38–41). Again, besides bacterial semen infections, the presence of HPV on the male partner is now considered a risk factor both for natural and assisted reproduction outcome (42, 43). In fact, it seems that when infection is present on sperm there is a negative effect on the ongoing pregnancy rate and live birth rate as well as an increase in the rate of miscarriage (44). Finally, anti-sperm antibodies (ASA) have been shown to reduce sperm motility, natural fertilization, and conception thus inducing couple fertility (45). They are frequently associated with semen infections and recently it has been demonstrated that ASA-positive men had lower rates of pregnancy and live births following IVF (46). Therefore, their presence should be considered in infertile couples. Finally, many other factors with more limited evidence are included, such as testicular torsion and microlithiasis.

### Assessment of Infertile Couples

The pathway to pregnancy in infertile couples is usually long and requires a high level of resilience owing to several factors including medical procedures, economic costs and psychological stress. In order to minimize the impact and to maximize the results, a well-integrated and multidisciplinary approach including all the different specialists of reproduction is necessary. In fact, it is mandatory to identify the most appropriate procedure with a gradual approach to both partners to obtain a precise diagnosis and the most effective therapeutic option, reducing invasive procedures that are not necessary.

For this reason, the first step should be a preliminary evaluation of both the male and female partner, drawing up the medical history and performing a clinical examination, taking into account the main issues listed in **Tables 2** and **3** for male and female respectively. In addition to the medical history and clinical examination, semen analysis, endocrine assessment, and ultrasound scanning should be performed in order to have a more comprehensive picture.

**Table 2:** First gynecological approach.

Medical history	Physical examination	Ultrasound
Iatrogenic causes Ovulation Ovaria reserve Age Metabolic factors Poliabortivity Lifestyle	BMI Hirsutism-hyperandrogenism Galactorreha Pelvic masses Cervical-vaginal disorders	PCO Ovarian Reserve Ovarian masses
Metrorrhagia VIP IUD Poliabortivity Malformations Previous surgery	Fibroids Malformations	Fibroids Malformations Endometrial polyps Endometrial thickness abnormalities
PID Dysmenorrhea/Dyspareunia Recurrent cystitis/vaginitis IUD Previous surgery	Sacto/hydrosalpinx Adhesions syndrom Endometriosis	Sacto/hydrosalpinx Adhesions syndrom Endometriosis

BMI, Body mass index; IUD, intrauterine contraceptive device; PCO, Polycystic ovary; PID, pelvic inflammatory disease; VIP, voluntary interruption of pregnancy.

**Table 3:** First andrological approach.

Medical history	Physical examination	Ultrasound	Sperm parameters
Family history of infertility Testicular trauma Previous infectious diseases Iatrogenic factors Endocrine diseases Using of anabolic steroids Puberty disorders Infertility with previous partner Occupational factors Lifestyle	Anthropometric measures Androgenization BMI/WC Testicular evaluation (morphology, size, position, masses presence) Varicocele/hydrocele Penis evaluation Gynecomastia	Testicular evaluation (morphology, size, masses presence) Varicocele/hydrocele Microlithiasis	Volume Total number pH Morphology Motility Vitality Swelling Antibody Viscosity

BMI, Body mass index; WC, waist circumference.

### Infertile Couple Pathway

**Figure 1:**
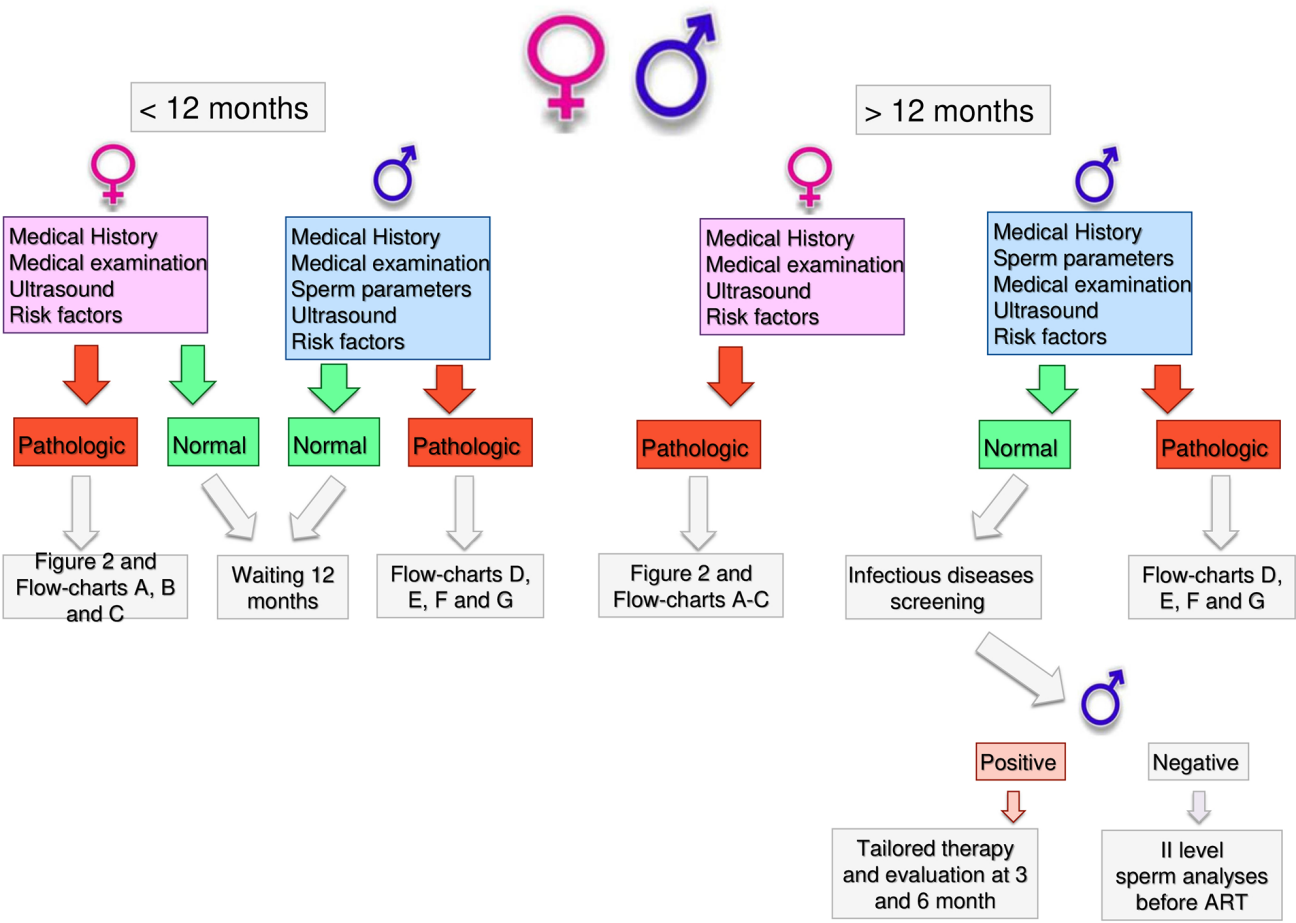
Infertile couple flow chart.

Health workers dealing with infertility should be able to guide couples through a clear, standardized and operative flow, reducing unnecessary burden on couples and optimizing the output of the diagnostic and treatment pathway. In **Figure 1**, we reported the results of an Italian consensus recommending a comprehensive approach to infertile couples. Based on WHO definition, couples should firstly be divided based on the time of offspring search (< or >12 months). The threshold chosen for the duration of pregnancy seeking is crucial and can impact on the prevalence of the disease, avoiding couples to undergo unnecessary diagnostic tests or to the risk of overdiagnosis (47). The WHO recommends preconception care between 3 and 6 months before trying for a baby because of its positive impact on maternal and child health outcomes (https://apps.who.int/iris/handle/10665/78067). As medical history, physical examination and ultrasound for women are part of routine pre-conception counselling, the gynecologic and whole health evaluation of the woman seeking pregnancy can be started independently of the diagnosis of infertility (http://www.undp.org/content/undp/en/home/librarypage/mdg/the-millennium-development-goals-report-2015.html). Possible factors involved in determining female infertility can be investigated as early as this stage (**Table 2**). For males, medical history, physical examination, sperm analysis, and ultrasound should be performed as shown in **Table 3** (48).

If no alteration or risk factors are highlighted by this first level examination, couples are recommended to have free intercourse waiting till 12 months. If any alteration or risk factor emerges, the affected partner should undergo further analyses as described below.

**Figure 2:**
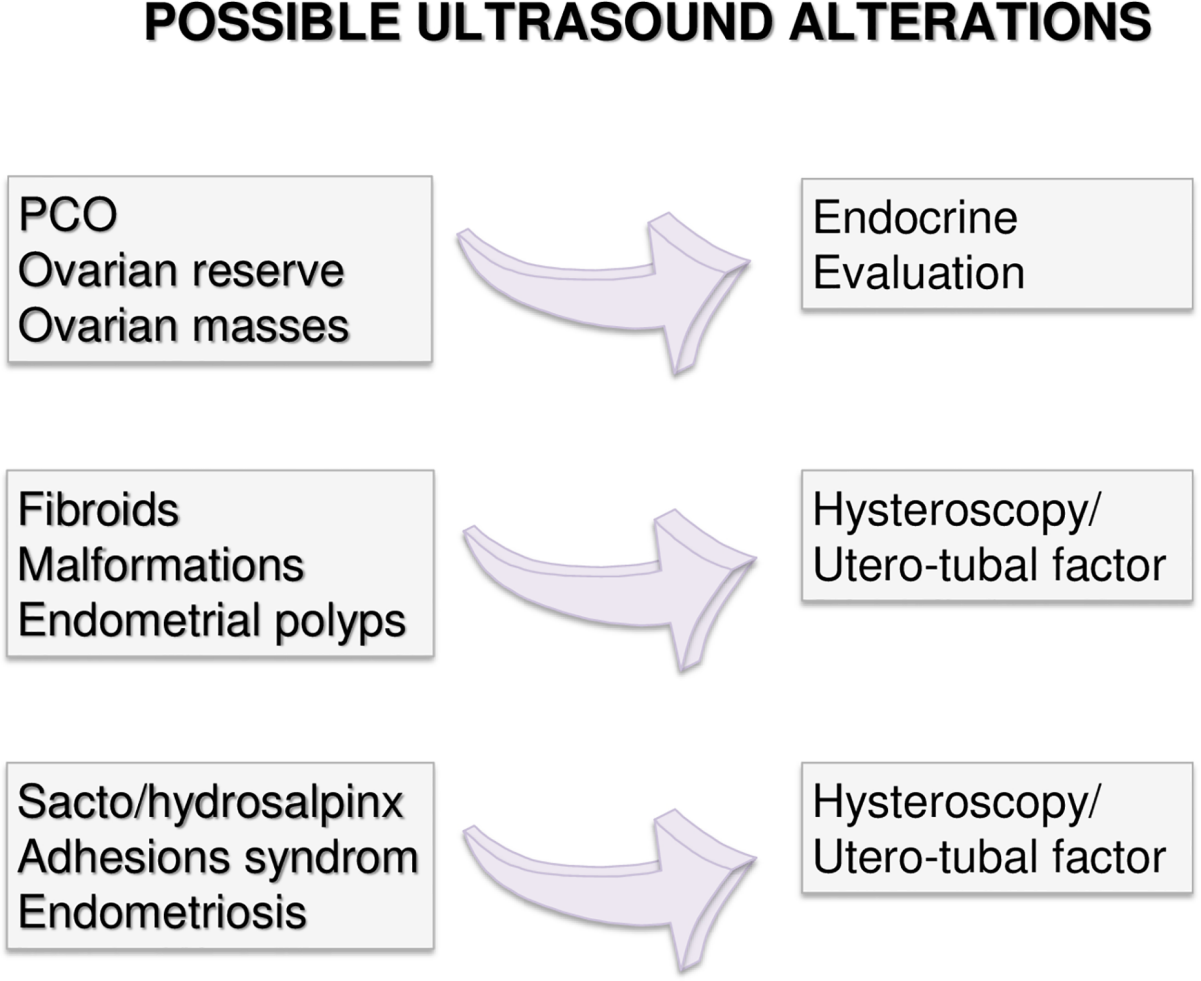
Possible alteration observable during trans-vaginal ultrasound.

**Figure 3:**
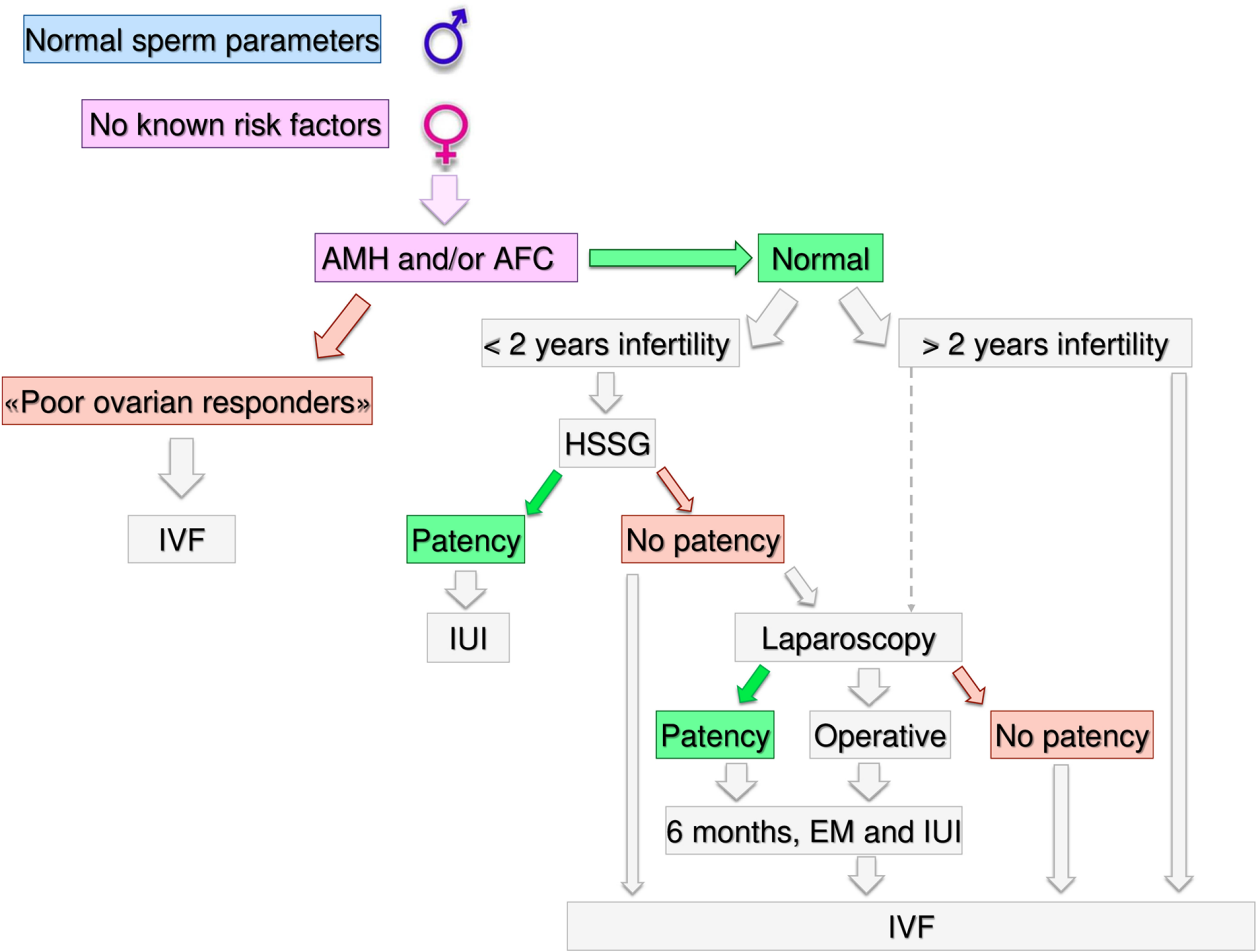
Gynecological specific flow chart: second level diagnostic investigation and possible therapeutic approach for woman with no known risk factors.

**Figure 4:**
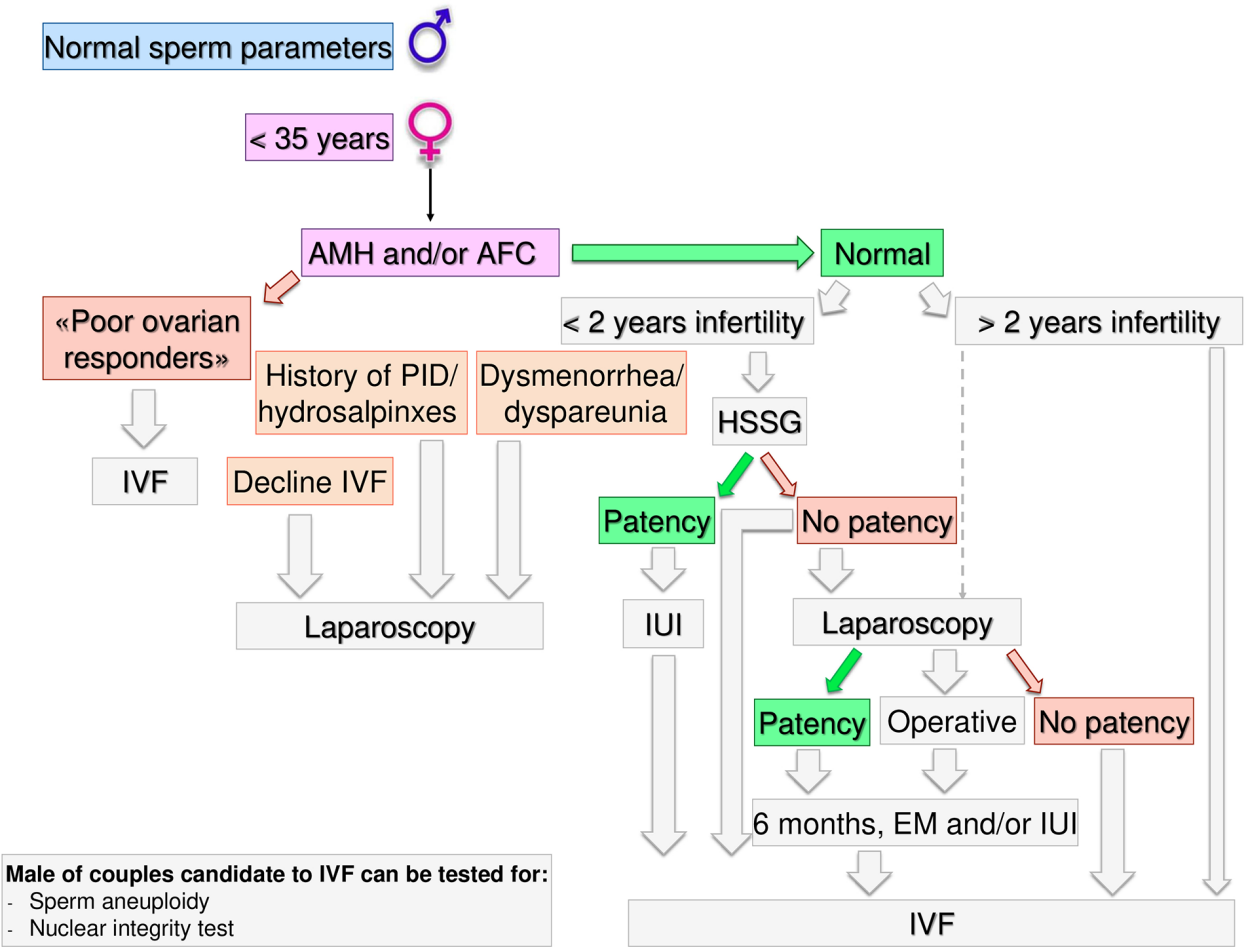
Gynecological specific flow chart: second level diagnostic investigation and possible therapeutic approach for woman with <35 years.

**Figure 5:**
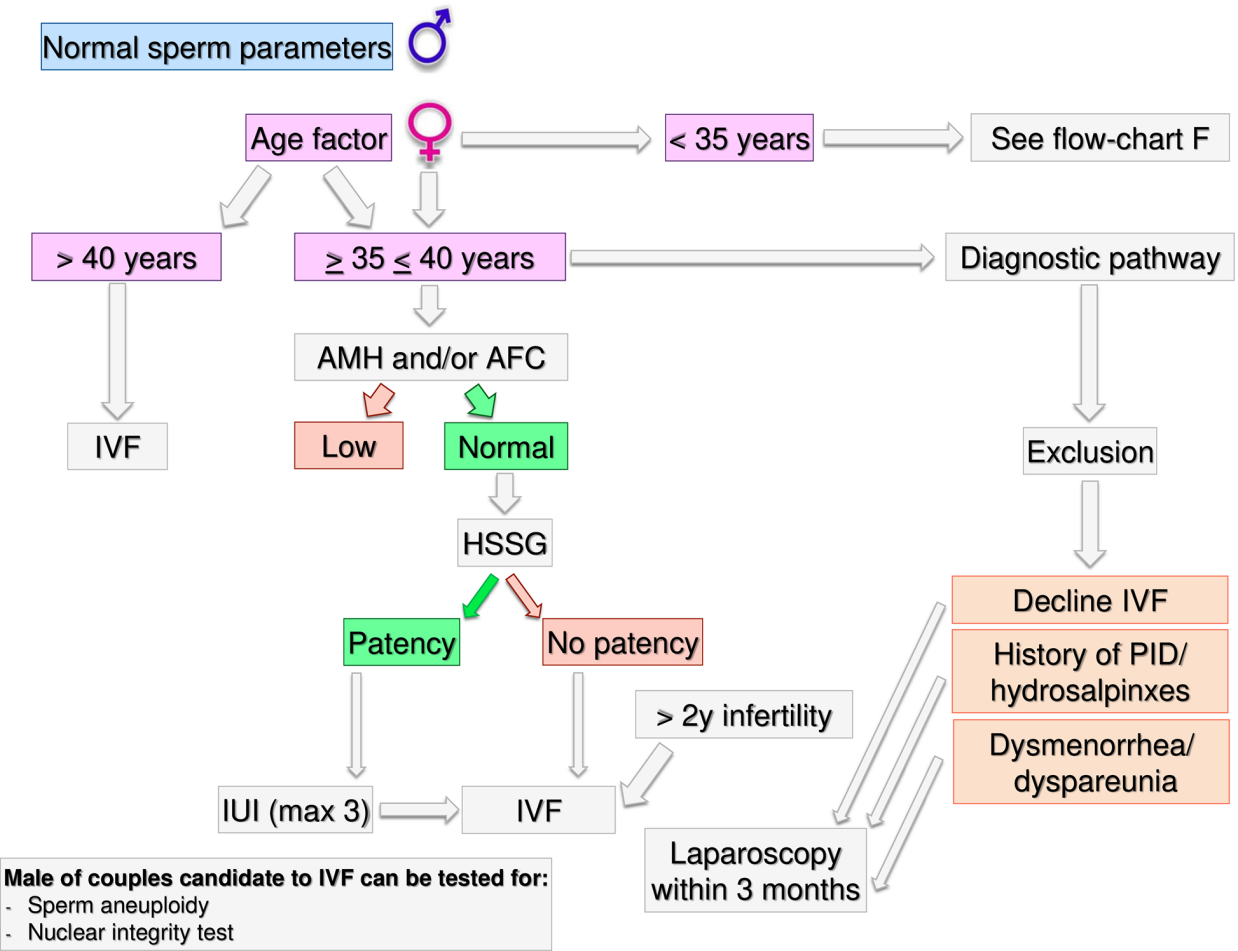
Gynecological specific flow chart: second level diagnostic investigation and possible therapeutic approach for woman with >35 years.

**Figure 6:**
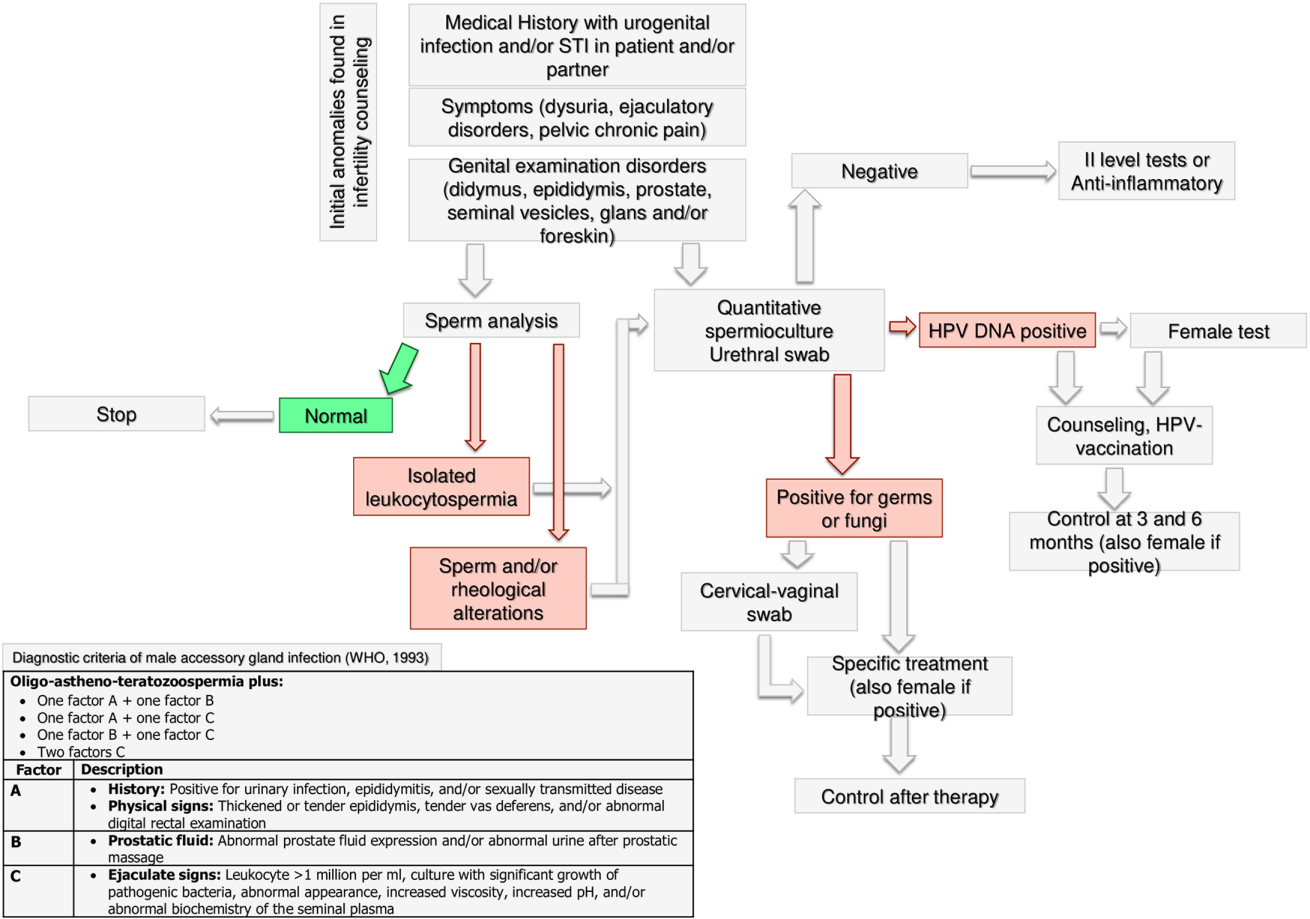
Andrological specific flow chart: microbiological assessment in male with anomalies found in infertility counseling.

**Figure 7:**
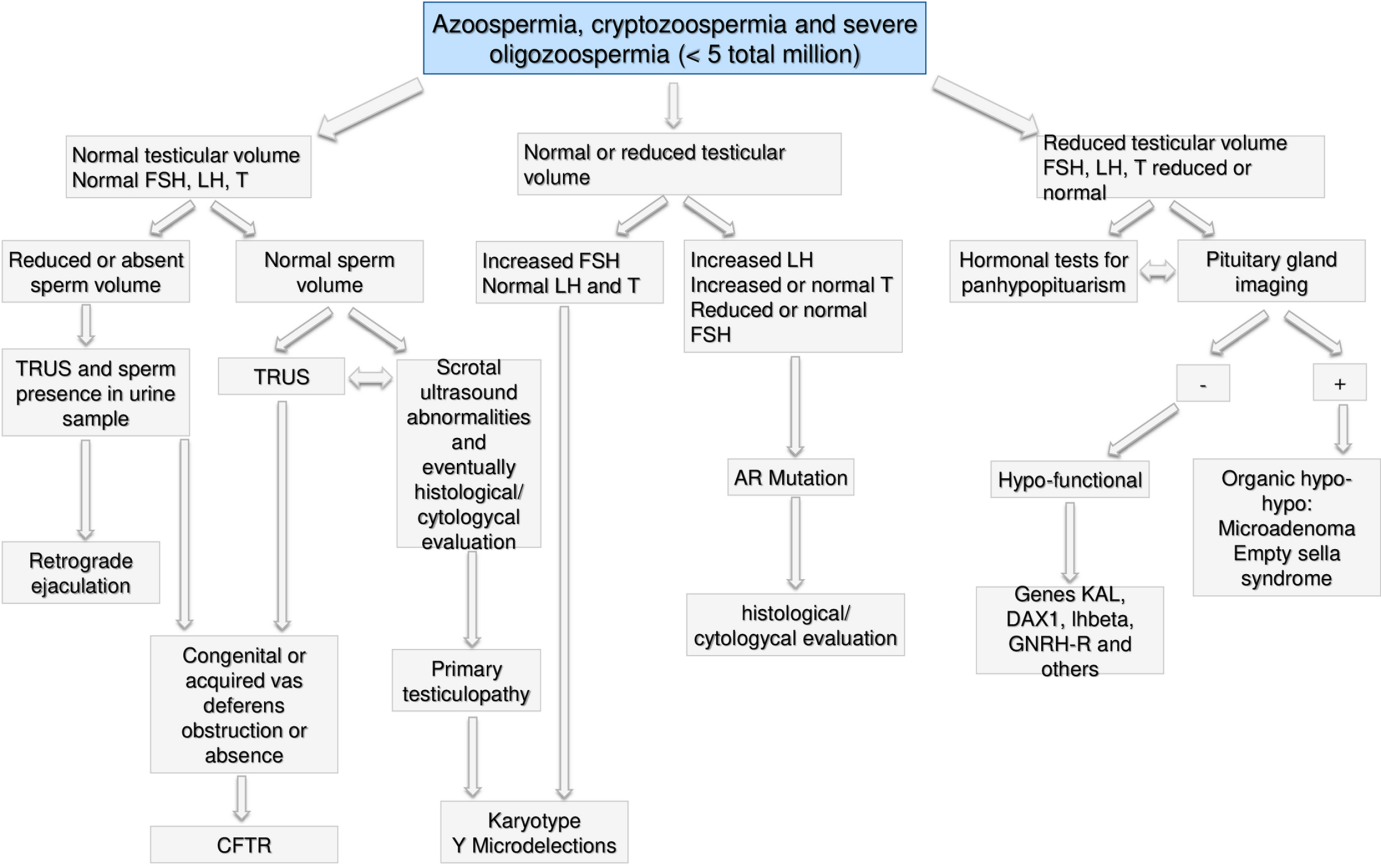
Andrological specific flow chart: diagnostic investigation for man with azoospermia, cryptozoospermia, and severe oligozoospermia (<5 total million).

**Figure 8:**
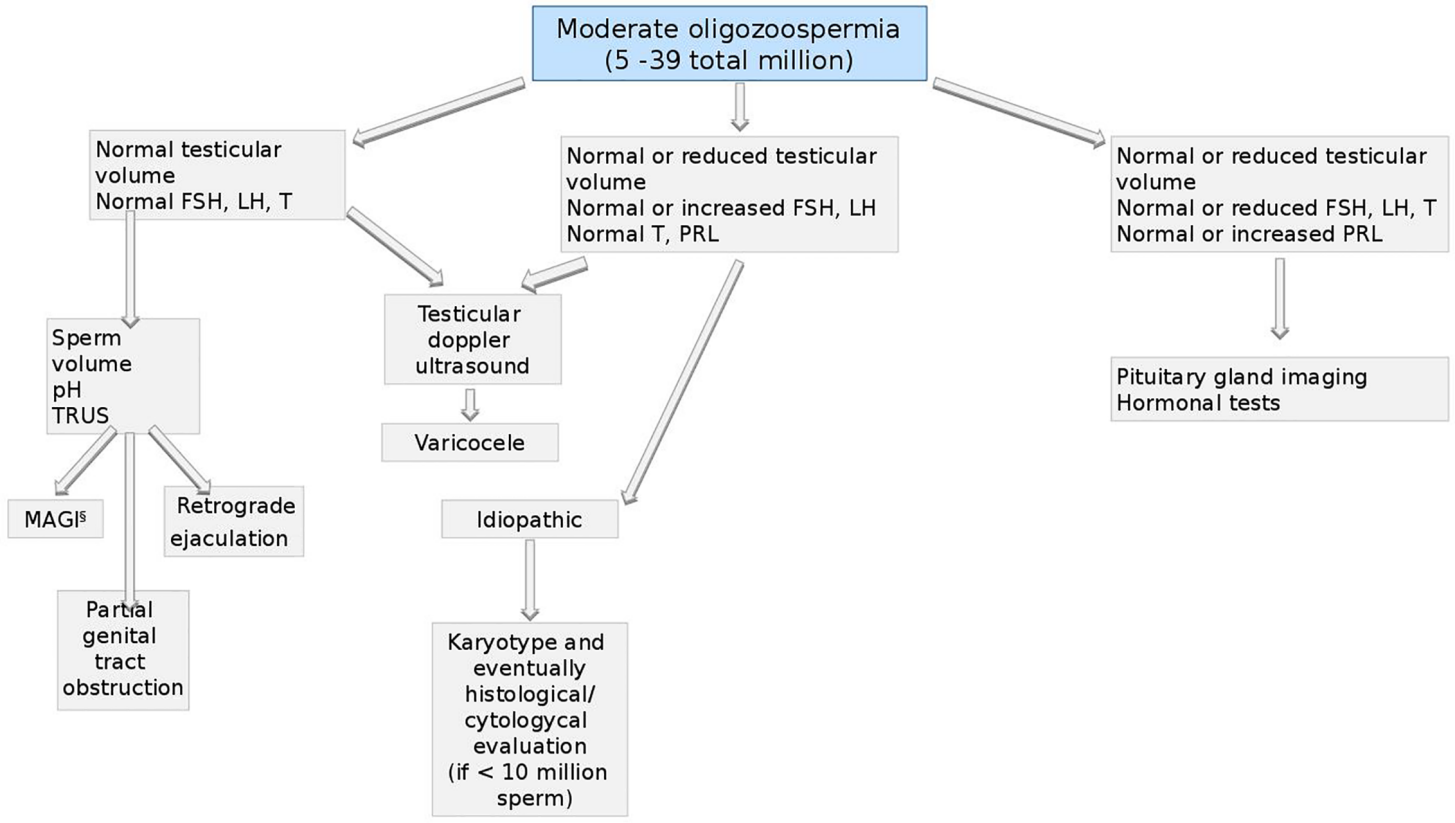
Andrological specific flow chart: diagnostic investigation for man with moderate oligozoospermia (5–39 total million).

**Figure 9:**
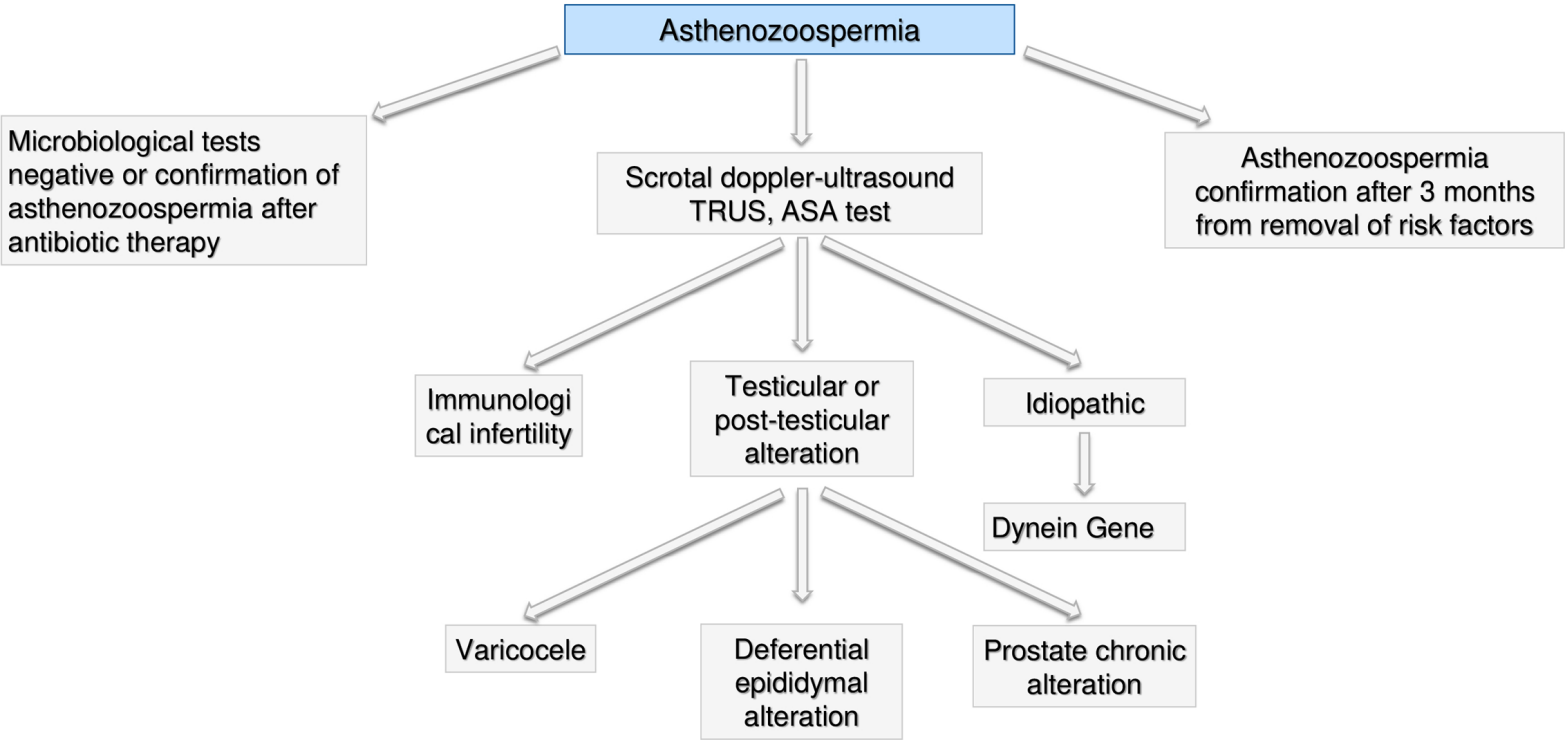
Andrological specific flow chart: diagnostic investigation for man with asthenozoospermia.

Couples with more than 12 months infertility, should undergo the same preliminary approach. If no alteration is highlighted in both partners, expectant management is acceptable. Indeed, no significant differences in live birth rates between expectant management and other interventions for unexplained infertility have been recently reported, at least excluding patients at poor prognosis of natural conception (49). However, alongside a waiting strategy, further diagnostic investigations are advisable: males should perform a comprehensive microbiological screening (see *Andrological Specific Flow-Charts*). If any infection is detected, it should be treated and checked after the end of treatment. On the other hand, if any alteration or risk factor emerges from the preliminary examination, the affected partner should undergo further analyses. In particular, targeted examination should be performed for women based on specific risk factors as alterations observed at trans-vaginal ultrasound (**Figure 2**) or clinical conditions (**Figures 3**–**5** explained below). For men, microbiology tests (**Figure 6**) or targeted flow charts (**Figures 7**–**9** explained below) should be followed based on sperm parameters.

### Gynecological Specific Flow Charts

#### Second Level Diagnostic Investigation

In a couple with > 12 months infertility, where the male partner has normal sperm parameters and woman has no known risk factors, second level investigations should start with the antral follicle count (AFC) and anti-mullerian hormone (AMH) dosage. If the woman was diagnosed as “poor responder”, the couple should be recommended to undergo in-vitro fertilization (IVF) independent of age (**Figure 3**). As the number and the quality of oocytes are directly related to the probability of obtaining a live birth through IVF, a waiting policy in these couples could be excessively penalizing if spontaneous conception was not obtained (50, 51). Indeed, the age-related decline in ovarian reserve has been shown greater in infertility patients than fertile women (52).

Likewise, women older than 40 years old should be directed to IVF independently from the ovarian reserve because in older women with no other risk factors the immediate access to IVF demonstrated superior pregnancy rates compared to other ART treatments (53). However, several key issues in reproductive physiology cannot be accurately investigated by means of the ordinary diagnostic work up. Thus, in women older than 40 years, unexplained infertility cannot be diagnosed easily and women encountering a physiological “age-related infertility” could be treated without a true indication (54). Although an immediate IVF strategy in these couples has theoretical advantages, there is no strong evidence in support of treatment versus a waiting policy (55).

If AFC and AMH are normal (i.e. patients cannot be defined as poor responders) further steps depend on the time of infertility (< or >2 years) and on female age. If the time of infertility is < 2 years, the management depends on the age: in women with less than 35 years (**Figure 4**), an hysterosalpingosonography (HSSG) should be performed. In case of tubal patency, it can be suggested intra-uterine injection (IUI) as first approach, as the chance of achieving a live birth are higher than expectant management (56). VF should be recommended after 3 IUI attempts (57), as the same chance to have a pregnancy by IVF (about 30% per attempt) are achieve at the seventh cycle of IUI (58). We do not consider all these attempts acceptable, as time to pregnancy and the couples’ compliance could be compromised after such numbers of failures. Furthermore, previous evidence suggests that the number of IUI attempts should be limited up to four (59–61).

If the HSSG highlights no tubal patency, a diagnostic and/or operative laparoscopy could be proposed, as laparoscopy in women with unexplained infertility may reveal previously undiagnosed pathologies that could require ART, and in those without abnormal surgical finding, ART does not improve pregnancy rate (62). If laparoscopy show tubal patency or it is possible to perform an operative laparoscopy, 6 months of free intercourses or IUI, can be suggested. If laparoscopy confirms tubal occlusions, the couple should be directed to IVF.

If a female age is between 35–40 years (**Figure 5**) HSSG could be performed. In case of tubal patency, IUI can be suggested as first approach. IVF should be recommended after 3 IUI attempts. If the HSSG highlights no tubal patency, IVF should be recommended. Independently from the ovarian reserve, in presence of hydrosalpinxes (often related to a PID history) a diagnostic and operative laparoscopy should be proposed before IVF (63–66). In case of dysmenorrhea/dyspareunia or others signs suggesting for mild endometriosis or IVF refusal (67, 68), a diagnostic laparoscopy within 3 months could be proposed, because undiagnosed or subtle pelvic abnormalities may be a significant cause of IVF failure (69).

If the time of infertility is >2 years and women age is <40 years, IVF should be proposed. Laparoscopy must be limited to few selected cases (IVF refusal, history of PID/hydrosalpinxes, dysmenorrhea/dyspareunia) after a careful risk benefit assessment. Male partner of couples candidate to IVF could be tested for sperm aneuploidy and nuclear integrity tests.

### Andrological Specific Flow Charts

#### Microbiological Assessment

The WHO guidelines for the management of male infertility include microbiological investigation and the diagnostic examinations of male accessory gland infection (MAGI) (70). MAGI comprise orchitis, epididymitis, vesiculitis, prostatitis, and urethritis, which are potentially reversible causes of male infertility (71). Following the WHO’s recommendations (70), medical history, physical examination and sperm analysis play a crucial role to suggest a microbiological assessment to the male partner of an infertile couple. In particular, leukocytospermia (leukocytes >1 million/ml), more frequently occurring in infertile patients compared to fertile men (72), deserves microbiological investigation, as suggested by the American Society for Reproductive Medicine (ASRM) Practice Committee (73).

There is a lack of consensus about the specific microbiological analysis that should be requested in infertile patients with MAGI. Although the WHO guidelines (70) indicate sperm culture, more recent research suggests that the urethral swab, which could more accurately detect intracellular microorganisms such as mycoplasmas (71, 74), may be useful. Accordingly, a systematic review with meta-analysis carried out on cohort and case-control studies found an association between *Mycoplasma hominis* and *Ureaplasma urealyticum* and male infertility, underlining the importance of their identification (75). If the culture is negative, the specialist could decide, case by case, both to treat the male with anti-inflammatory or to request second level tests. In the case of positive culture, the female partner should also be tested and tailored treatment should be prescribed to patients with infection. A microbiological re-evaluation at the end of the treatment may be useful because of the relatively high persistence rate at the end of the antibiotic treatment (71).

The microbiological assessment of infertile couples should include the search for Papillomavirus (HPV) DNA, especially in case of current or anamnestic presence of condylomatosis, or in recurrent pregnancy loss. Accordingly, HPV infection shows a significantly higher prevalence in infertile patients compared to fertile men (20.4% vs. 11.4%), as indicated by a meta-analysis (76). Also, results from 5203 men revealed significantly worse conventional sperm parameters in HPV-positive than HPV-negative patients, with the motility being the parameter more strongly associated with HPV-infection. Importantly, a significantly higher miscarriage rate has been reported in HPV-positive patients compared to controls (77). Finally, if the HPV DNA test is positive, both male and female should be counseled for possible HPV vaccination and followed-up at 3 and 6 months after counseling.

#### Azoospermia, Cryptozoospermia, and Severe Oligozoospermia (< 5 Total Million)

The first step for men with azoospermia, cryptozoospermia and severe oligozoospermia (<5 total million sperm), should be the assessment of testicular volume and sex hormones levels (48, 73, 78).

If both, testicular volume and FSH, LH, and total testosterone (T) are normal, further analyses are performed based on semen volume: on one hand, if it is reduced or absent, a trans-rectal ultrasound (TRUS) and the search of sperm in the urine sample should be performed to evaluate the possibility of retrograde ejaculation (79–82) or vas deferens obstruction. In the latter case, the CFTR gene mutations should be investigated (83). On the other hand, if the semen volume is normal, TRUS could highlight genital tract obstruction and vas deferens pathologies and should be investigated as above described (84). At the same time scrotal ultrasound should exclude epididymal abnormalities (85, 86). If no alteration is found, histological/cytological evaluation may be performed to highlight spermatogenic function, distinguishing obstructive forms from primary testiculopathy (87, 88). In case of spermatogenic impairment, it makes sense to perform genetic screening for karyotype and Y microdeletions (89–91).

If the testicular volume is normal or reduced, hormonal levels lead to further investigation: in case of increased FSH and normal LH and testosterone levels, genetic screening for karyotype and Y microdeletions should be performed (92) and histological/cytological evaluation procedure could be performed aimed to clarify the tubular status. When LH is increased, testosterone normal or increased and FSH normal or reduced, the genetic screening for androgen receptor (AR) mutations should be performed (93) and also in this case, histological/cytological evaluation option may be considered.

Lastly, if the testicular volume is reduced, FSH and LH and testosterone are normal or reduced, pituitary-testicular axis should be assessed by both, hormonal tests for hypopituitarism and pituitary imaging to assess the abnormalities (94). In addition, if pituitary imaging doesn’t reveal any alteration, genetic screening for KAL, DAX1, LHbeta, and GNRH-R gene mutations should be performed (95–97).

#### Moderate Oligozoospermia (5–39 Total Million)

Also for men with moderate oligozoospermia (from 5 to 39 total million sperm), the first approach should be the assessment of testicular volume and hormonal asset (48, 73, 78).

If both, volume and hormones are normal, testicular doppler ultrasound should be performed aimed to exclude the presence of varicocele (98–100). Also based on semen volume, pH, and TRUS, four conditions should be considered: MAGI (see the section *Microbiological Assessment—* **Figure 6**), partial genital tract obstruction (101), retrograde ejaculation, and idiopathic oligozoospermia (102).

In the last scenario, a genetic screening for karyotype should be performed (92) and, in case of sperm count below 10 million, histological/cytological evaluation, could highlight the cause of oligozoospermia (88, 103).

In presence of normal or reduced testicular volume, normal or increased FSH and LH and normal T and PRL, the presence of varicocele or idiopathic infertility should be investigated as described above (99, 100).

Finally, if testicular volume, FSH, LH and T are normal or reduced and PRL is normal or increased, pituitary-testicular axis should be assessed by both, imaging and further hormonal test for hypopituitarism as previously described (94) (see **Figure 7**).

#### Asthenozoospermia

In presence of isolate asthenozoospermia, first of all genital tract infections should be excluded (see the section *Microbiological Assessment—* **Figure 6**) and a further semen analysis should be performed after 3 months from the end of treatment to re-evaluate such condition. If confirmed, a scrotal doppler-ultrasound, a TRUS and test for anti-sperm antibodies (ASA) test should be performed (104). Based on the results, we could face three scenarios: i) immunological infertility (105); ii) testicular or post-testicular infertility, including epididymal-deferential alterations, prostate chronic abnormalities or varicocele (106, 107) and iii) idiopathic infertility. In the case of idiopathic infertility, mutation of dynein gene should be investigated (108, 109). In case of presence of possible environmental or lifestyle causes of semen alteration, asthenozoospermia should be confirmed after 3 months from removal of risk factors (frequent saunas, smoking, work activities, tight sportswear) (108, 110, 111).

## Conclusions

This expert opinion is an attempt to create a comprehensive, clear, standardized and personalized approach to infertile couples considering their health and wellness of offspring conceived. It contains practical recommendations useful for health workers dealing with reproductive health and to the research community. Considering the dynamism and the continuous evolution in the setup of diagnostic tools, this expert opinion provides just a general informed debate intending to stimulate further discussions among all those interested in the scientific, social, and ethical aspects of ART and to provide some guidance and clarification for ongoing discussion.

## The Infertilitaly Group

*Andrologists*: Angela Alamo, Aldo Eugenio Calogero, Rossella Cannarella, Nicola Caretta, Francesco Cargnelutti, Mario Ciletti, Laura Cimino, Stefano Colangelo, Michele Compagnone, Rosita A. Condorelli, Christian Corsini, Alessandro Dal Lago, Giuseppe Defeudis, Antonella Di Mambro, Alberto Ferlin, Carlo Foresta, Giorgio Franco, Mariagrazia Gallo, Andrea Garolla, Marco Ghezzi, Daniele Gianfrilli, Giuseppe Grande, Michele Guidotti, Sandro La Vignera, Maria Agnese Latino, Andrea Lenzi, Emanuele Licata, Francesco Lombardo, Francesco Lotti, Giovanni Luca, Massimo Manno, Marco Marasco, Fernando Mazzilli, Rossella Mazzilli, Maurizio Merico, Domenico Milardi, Laura M. Mongioì, Pierfrancesco Palego, Alessandra Petrozzi, Damiano Pizzol, Rocco Rago, Francesco Romanelli, Riccardo Selice, Lee Smith, Umberto Valente.

*Gynecologists:* Anna Biasioli, Andrea Borini, Andrea Roberto Carosso, Claudio Castello, Maria Elisabetta Coccia, Cristofaro De Stefano, Lino Del Pup, Stefano Facchin, Andrea Gallinelli, Gianluca Gennarelli, Maurizio Guido, Assunta Iuliano, Giovanni Battista La Sala, Irene Ladisa, Antonio Lanzone, Stefano Palomba, Fedro Alessandro Peccatori, Fabio Perricone, Stefania Piccolo, Alberto Revelli, Francesca Rizzello, Chiara Romanelli, Francesca Salvagno, Sergio Schettini, Paolo Scollo, Francesco Tomei, Filippo Maria Ubaldi, Francesca Vasoin, Alessandra Vucetich.

*Oncologist:* Giovanni Codacci-Pisanelli.

*Biologists and Embryologists*: Attilio Anastasi, Elisabetta Baldi, Alberto Bottacin, Francesco Capodanno, Tania Carlini, Sara Corrò, Ilaria Cosci, Giovanni Coticchio, Lucia De Santis, Fabiana Faja, Elena Marcazzan, Massimo Menegazzo, Alessio Paffoni, Donatella Paoli, Nicola Passerin, Alessandra Patimo, Marianna Pelloni, Adriano Presciutti, Laura Rienzi, Jessica Sebellin, Riccardo Talevi, Chiara Tempio.

*Geneticists:* Ivana Antonucci, Savina Dipresa, Valentina Gatta, Antonio Novelli, Liborio Stuppia.

*Microbiologists:* Valeria Meroni, Guido Scalia, Alessandra Sensini.

## Author Contributions

All authors contributed to the article and approved the submitted version.

## Conflict of Interest

The authors declare that the research was conducted in the absence of any commercial or financial relationships that could be construed as a potential conflict of interest.
